# 7-Methylguanosine Modifications in Transfer RNA (tRNA)

**DOI:** 10.3390/ijms19124080

**Published:** 2018-12-17

**Authors:** Chie Tomikawa

**Affiliations:** Department of Materials Science and Biotechnology, Graduate School of Science and Engineering, Ehime University, Bunkyo 3, Matsuyama, Ehime 790-8577, Japan; tomikawa.chie.mm@ehime-u.ac.jp; Tel./Fax: +81-89-927-9947

**Keywords:** RNA modification, tRNA methyltransferase, tRNA modification, methylase

## Abstract

More than 90 different modified nucleosides have been identified in tRNA. Among the tRNA modifications, the 7-methylguanosine (m^7^G) modification is found widely in eubacteria, eukaryotes, and a few archaea. In most cases, the m^7^G modification occurs at position 46 in the variable region and is a product of tRNA (m^7^G46) methyltransferase. The m^7^G46 modification forms a tertiary base pair with C13-G22, and stabilizes the tRNA structure. A reaction mechanism for eubacterial tRNA m^7^G methyltransferase has been proposed based on the results of biochemical, bioinformatic, and structural studies. However, an experimentally determined mechanism of methyl-transfer remains to be ascertained. The physiological functions of m^7^G46 in tRNA have started to be determined over the past decade. For example, tRNA m^7^G46 or tRNA (m^7^G46) methyltransferase controls the amount of other tRNA modifications in thermophilic bacteria, contributes to the pathogenic infectivity, and is also associated with several diseases. In this review, information of tRNA m^7^G modifications and tRNA m^7^G methyltransferases is summarized and the differences in reaction mechanism between tRNA m^7^G methyltransferase and rRNA or mRNA m^7^G methylation enzyme are discussed.

## 1. Introduction

**Figure 1:**
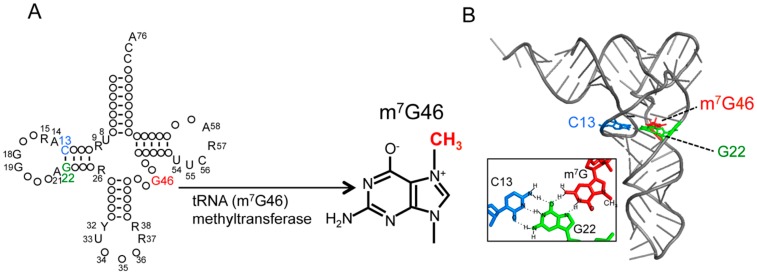
tRNA m^7^G46 methyltransferase methylates the N7-atom of guanine at position 46 in tRNA and forms m^7^G46. (**A**) The secondary structure of tRNA is presented in cloverleaf form. Conserved nucleotides are depicted as follows: adenosine, A; guanosine, G; cytidine, C; uridine, U; purine, R; pyrimidine, Y. tRNA (m^7^G46) methyltransferase transfers a methyl-group to the N7-atom of guanine at position 46 in tRNA and forms 7-methylguanine. (**B**) The L-shaped structure of tRNA is presented. The m^7^G46 forms a tertiary base pair with the C13-G22 base pair in the L-shaped tRNA structure. In the stick model, only atoms are visible and charge is invisible.

Transfer RNA, which is one of the classical non-coding RNAs, functions as an adaptor molecule supplying amino acids to ribosomes according to the codon of the mRNA. It is important that tRNA forms a precise L-shape structure for its full function [1] and this requires tRNA modification. More than 100 different modified nucleosides have been reported to date and found throughout the different families of RNA molecules [2,3]. tRNA in particular is the most heavily modified [3,4]. The modified nucleosides include thiolation, deamination, isomerization conversion of uridine to pseudouridine, or the combination of several modifications. Of the modification, methylation is the most abundant. This modification encompasses 1-methyladenosine (m^1^A), 5-methyluridine (m^5^U), 5-methylcytidine (m^5^C), 1-, 2-, or 7- position methylation of G (m^1^G, m^2^G, m^7^G), 2’-*O*-methylation of ribonucleoside (Nm), and others [3,5,6]. The most widely prevalent tRNA methylation is S-adenosyl-L-methionine (AdoMet)-dependent methylation by AdoMet dependent methyltransferases. 7-methylguanosine (m^7^G) is one of the most conserved modified nucleosides and is common in eubacteria, eukaryotes [4], and a few archaea [7]. Even in psychrophiles which have low levels of modified nucleoside content, m^7^G has been found in addition to dihydrouridine (D), pseudouridine (Ψ), and m^5^U [8]. Additionally, m^7^G is present in introns containing pre-tRNA together with *N*^2^,*N*^2^-dimethylguanine (m^2^_2_G), Ψ and m^1^A [9]. For this reason, it is thought that m^7^G is generated immediately after the transcription. m^7^G is most frequently located at position 46 in the tRNA variable region, and forms a tertiary base pair with C13-G22 in the three-dimensional core of tRNA (The nucleotide positions in tRNA are numbered, according to the reference [4].) [10,11,12]. m^7^G has no (net) charge under physiological conditions, but is positively charged in position 46 in tRNA via hydrogen bonding to bases G22 and C13 [13,14,15] (Figure 1). Thus, 7-methylation of m^7^G produces a site-specific electrostatic charge within the tRNA structure [15]. The tertiary base pair of the m^7^G46-C13-G22 is considered to contribute to stabilization of the tRNA three-dimensional core.

**Figure 2:**
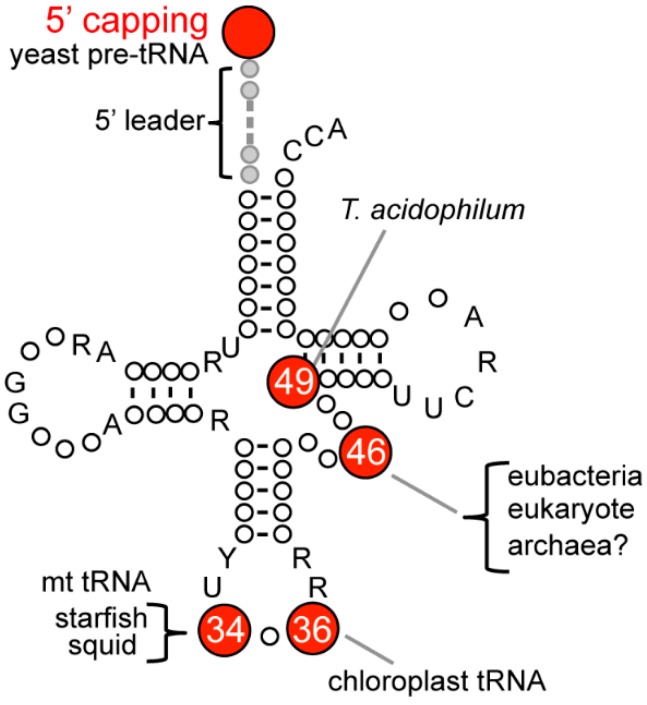
Positions of the m^7^G modification in tRNA. The numbers in circles indicate the positions of m^7^G in tRNA.

There are some examples where m^7^G is found in positions other than at position 46 (Figure 2, Table 1). Chloroplast tRNA^Leu^(UAG) from *Chlamydomonas reinhardtii* has m^7^G at position 36 in the tRNA anticodon [16]. In animal mitochondria in which there is deviation from the universal genetic code, m^7^G is present at position 34 in the anticodon of mitochondorial (mt) tRNA^Ser^(GCU) of starfish, *Asterina amurensis* [17], and squid, *Loligo bleekeri* [18]. Furthermore, the D-arms of the mt tRNA^Ser^(m^7^GCU) in starfish and squid have an unusual secondary structure, and the mt tRNA^Ser^(m^7^GCU) recognizes not only the serine codons AGU and AGC but also the unusual serine codons AGA and AGG. Thus, m^7^G can make base-pairing with the four basic bases of A, U, G, and C [19]. With respect to m^7^G in archaeal tRNA, in 1991, Edmonds et al. reported that tRNA mixtures from some archaea contain m^7^G nucleoside; however, the position(s) in tRNA has remained unidentified [7]. More than twenty years later, it was demonstrated that the presence of the m^7^G modification in a thermo-acidophilic archaeon, *Thermooplasma acidophilum* at the novel, irregular position 49 in class II tRNA^Leu^ [20]. However, the gene encoding the methyltransferase for m^7^G49 in tRNA has not been identified thus far.

The 7-methylguanosine modifications occur not only in tRNA, but also in other RNA species such as mRNA, ribosomal RNA (rRNA), small nuclear RNA (snRNA), and small nucleolar RNA (snoRNA). The 5′ terminus of eukaryotic mRNA is blocked by m^7^G^5′^ppp^5′^N cap structure [33,34], and gene of cap-m^7^G methyltransferase (Abd1) is essential in *Saccharomyces cerevisiae* [35]. Surprisingly, recent work has detected a cap structure in tRNA. In yeast, an m^7^G cap structure is found at the 5′ termini of pre-tRNA bearing 5′ leader sequences (Figure 2). The capped pre-tRNAs accumulate due to inhibition of 5′ exonucleases activities and protect pre-tRNAs from 5′-exonucleolytic degradation during maturation [31]. 7-methylguanosine is observed in 16S rRNA of aminoglycoside-producing Actinobacteria, including *Streptomyces tenebrarius* and *Micromonospora purpurea*. The m^7^G modification is at position G1405 in the 16S rRNA and has an aminoglycoside resistance activity [36,37,38].

Since Holley et al. determined the sequence of yeast tRNA^Ala^ in 1965 [39], various tRNA sequences have been reported, and the presences of modified nucleosides in tRNA have been revealed. Also, the technical method of m^7^G detection has a history as well as tRNA sequencing. Initially, Wintermeyer and Zachau described a specific chemical method in which m^7^G detection is achieved via aniline-induced cleavage of the tRNA strand by β-elimination after additional treatment under alkaline conditions or after its reduction by sodium borohydride (NaBH_4_) in tRNA [40,41]. Both reduced m^7^G and its degradation products are susceptible to hydrolysis of the *N*-glycoside bond with subsequent chain scission by the β-elimination. By combining the aniline cleavage method and the Donis-Keller-method, which uses ribonucleases [42,43], it is possible to identify the position of m^7^G in tRNA. These methods are utilized for m^7^G detection not only in tRNA but also in rRNA [44]. In the Donis-Keller-method, RNsase T1 is used for detection of guanosine position. Although RNase T1 specifically digests the phosphodiester bond of guanosine-3′ phosphate in ribonucleic acid and ribonucleotide, the 7-methyl modification of guanosine prevents RNase T1 cleavage. Additionally, in pre-tRNA containing m^7^G at the 5′-end of the acceptor stem, the m^7^G modified nucleoside absolutely prevents cleavage by M1 RNA, the catalytic RNA subunit of RNase P [45]. Inhibition by m^7^G is thought to be because the approach of positive magnesium ion as a cleavage agent becomes impossible due to the positive charge of m^7^G. In addition, antibodies which specifically target *N*^6^-methyladenine (m^6^A) and m^7^G were prepared by immunization of rabbits with nucleoside conjugates of bovine serum albumin (m^6^A-BSA, m^7^G-BSA) [46]. Both the anti-m^7^G and anti-m^6^A antibody adsorbents became a tool for fractionation of oligonucleotides and nucleic acids. Currently, not only the m^7^G modification but also a variety of other modified nucleosides can be detected more quickly and accurately by mass spectrometry or high performance liquid chromatography [47]. Moreover, only recently, Motorin and co-authors reported a deep sequencing method named AlkAniline-Seq for the detection of m^7^G in RNA at single nucleotide resolution. AlkAniline-Seq is exploited the generation of abasic sites by alkaline hydrolysis and aniline cleavage. The method allows for sensitive m^7^G detection of total RNA from cells [48].

The enzymatic activity of tRNA (m^7^G46) methyltransferase was initially confirmed in cell extracts from *Escherichia coli* [49] and has been purified more than 1000-fold [50]. Enzymatic activities have also been detected from *Salmonella typhimurium* [51,52], *Thermus flavus* [53], *Xenopus laevis* [54], humans [55], and plants [9]. The m^7^G46 modification is generated by tRNA (m^7^G46) methyltransferase (tRNA (guanine-7-)-methyltransferase, EC 2.1.1.33; TrMet (m^7^G46)) [3,56] The gene encoding tRNA (m^7^G46) methyltransferase was first identified in yeast and was shown to be composed of two protein subunits Trm8 and Trm82 encoded by YDL201w and YDR165w, respectively [28]. Although Trm8 is the catalytic subunit, formation of a complex with Trm82 is required for the enzymatic activity [57]. Following this report, eubacterial genes have also been identified as *trmB*, whose classical name is *yggh*, in *E. coli* [58], *Aquifex aeolicus* [23], and *Bacillus subtilis* [27] (Table 1).

In this review, information of tRNA m^7^G modifications and tRNA m^7^G methyltransferases since m^7^G was discovered in tRNA is summarized, and the differences in reaction mechanism between tRNA m^7^G methyltransferase and rRNA or mRNA m^7^G methylation enzyme are discussed.

## 2. Structural Analyses and Catalytic Mechanisms of m^7^G Methyltransferases

Structural studies of tRNA modification enzymes can be informative both for the specificity and catalytic mechanism of the enzymes. Furthermore, the combination of structural and biochemical analysis data allows comparison of reaction mechanisms from different species. This makes it possible to infer information about molecular evolution. The crystal structures of tRNA (m^7^G46) methyltransferase from *B. subtilis* [27], *E. coli* [21], *Streptococcus pneumoniae* (PDB: 1YZH), and *S. cerevisiae* [29] have been reported (Table 1). X-ray crystallographies of TrmB and Trm8 have revealed classic class I AdoMet-dependent methyltransferase structures. These AdoMet-dependent tRNA MTases belong to two protein super families, which are structurally and phylogenetically unrelated, namely, the Rossmann fold MTases (RFM) and SPOUT MTases (SpoU and TrmD) [59]. SPOUT MTases have a deep trefoil knot structure which forms the catalytic site and the cofactor-binding pocket [59,60,61]. TrmB and Trm8 belong to the RFM family of MTases [29,58].

### 2.1. Eubacterial tRNA m^7^G46 Methyltransferases (TrmB)

**Figure 3:**
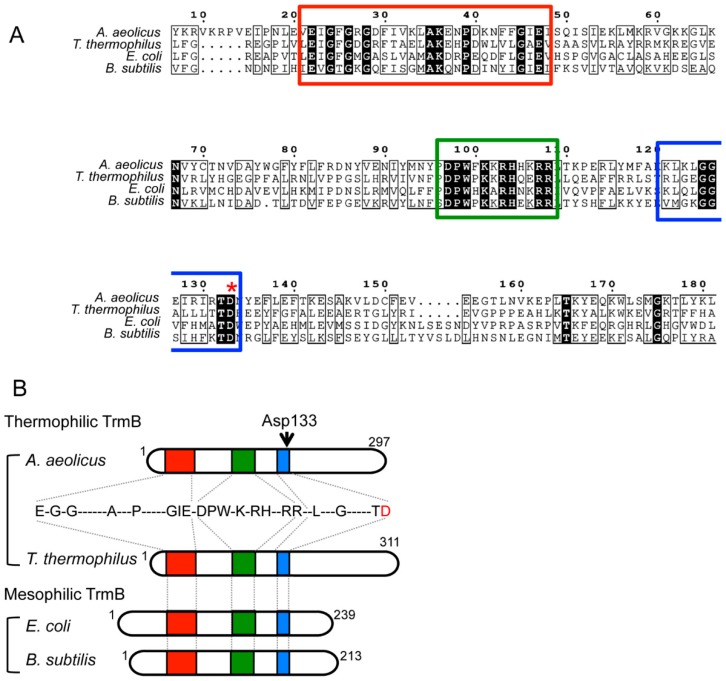
Comparison of thermophilic and methophilic TrmB. (**A**) Sequence alignment of TrmB. Conserved regions are highlighted in three colored squares (red, green, blue). Asp133 is indicated by an asterisk. (**B**) Thermophilic and mesophilic TrmB proteins are illustrated schematically. The three colored regions correspond to the amino acid sequences in panel A. Thermophilic TrmB has a distinct long C-terminal region. Asp133 is highlighted with an arrow, and the red D corresponds to the Asp133.

**Figure 4:**
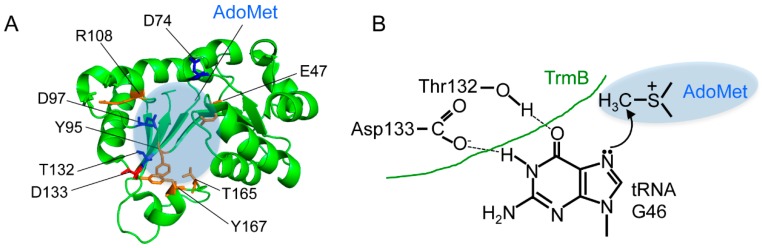
The amino acid residues for AdoMet binding and hypothetical reaction mechanism of TrmB. (**A**) The amino acid residues (E47, Y95, R108, T165 and Y167) involved in AdoMet binding (orange) are indicated on the catalytic domain of *B. subtilis* TrmB structure (PDB: 2FCA). The hypothetical catalytic center (grey shading), Asp133 (red) and the other important amino acid residues (D74, D97 and T132; blue) are highlighted. (**B**) Hypothetical reaction mechanism of eubacterial TrmB proteins is drawn.

Eubacterial TrmB exists as either a single subunit or a homodimer. TrmB of *B. subtilis* and *S. pneumoniae* has a dimeric structure both in solution and in the crystal form. However, dimerization does not seem to be a common feature of the enzymes, as the interface is not conserved and the TrmB enzymes of *E. coli* and *A. aeolicus* are monomeric. Analysis of TrmB activity using mutant proteins based on bioinformatic studies has revealed residues important for function [22]. The thermophilic TrmB has a longer C-terminal region compared to the methophilic TrmB [23,24] (Figure 3B). It has been reported that the C-terminal region is required for protein stability at high temperatures and contributes to the selection of the precise guanine nucleotide (i.e., G46) to be modified [24]. Alanine substitution of Arg287 in the long C-terminal region considerably reduces the methyltransfer activity. Thus, a part of the C-terminal region may make contact with tRNA. In contrast, the methophilic TrmB proteins from *E. coli* and *B. subtilis* have a long N-terminal region, and it has been shown that Arg26 in *E. coli* TrmB is involved in activity [22]. Furthermore, when Asp133 is replaced by alanine or asparagine in *A. aeolicus* TrmB, the methyltransfer activity is completely lost [24]. The aspartic acid residue is highly conserved in both TrmB and Trm8. Therefore, the Asp133 of *A. aeolicus* TrmB is considered as the catalytic center. Furthermore, in a docking model of guanine and the Trm8–Trm82 complex, the corresponding aspartic acid has the same positional relationship [29]. For this reason, it is suggested that Asp133 of *A. aeolicus* TrmB captures the G46 base of tRNA. Mutagenesis study has shown that Asp133 and several amino acid residues may contribute to AdoMet binding (Figure 4A). Taken together, it has been proposed a hypothetical mechanism for TrmB in which the carboxyl group of Asp133 captures the proton of N–H of the guanine base and the N7 atom of the guanine base itself attacks the methyl group in AdoMet [25] (Figure 4B). A hypothetical catalytic mechanism for TrmI, which is a methyltransferase for the N1 atom of adenosine at position 58 in tRNA, has also been proposed [62]. In this mechanism, the N1 atom causes a nucleophilic attack on the methyl group of AdoMet. Since the reactivity of nitrogen atoms in the bases is generally higher than the reactivity of carbon and oxygen atoms [56], in some methyl group transfer reactions, the nitrogen atom itself seems to directly attack the methyl group of AdoMet.

### 2.2. Heterodimeric tRNA m^7^G Methyltransferase of Yeast (Trm8/Trm82)

Yeast Trm8/Trm82 proteins are unrelated and have no homology to each other. This two-protein complex is conserved in eukaryotes. The homologous human proteins METTL/WDR4 complement Trm8/Trm82 in yeast [28]. Structural analysis demonstrated that Trm82 protein is a member of the WD fold family. Trm82 adopts a β propeller fold and contains seven blades [29]. In yeast, two-subunit tRNA methyltransferases have been identified. Trm11-Trm112 (m^2^G10), Trm7-Trm732 (2′-*O*-methylcytidine (Cm32)), Trm7-Trm734 (Nm34), Trm9-Trm112 (5-methoxycarbonylmethyluridine (mcm^5^U34) and 5-methoxycarbonylmethyl-2-thiouridine (mcm^5^s^2^U34)), and Trm6-Trm61 (m^1^A58) exist (Modified nucleosides are in parentheses) [63,64,65,66,67]. Trm112, which is a small protein and is conserved in all three domains of life, interacts and activates four methyltransferases, Bud23, Trm9, Trm11 and Mtq2. The targets of these methyltransferase are components of different parts of the translation machinery, namely, rRNA, tRNAs, and release factors [68]. These complexes are composed of a methyltransferase catalytic subunit and a noncatalytic subunit. The noncatalytic subunits are involved in stabilizing the catalytic subunit or activating and fine tuning the activity. By using a wheat germ cell-free system, Trm8 and Trm82 from their respective mRNAs were expressed. Separate expression of Trm8 and Trm82 proteins and their subsequent mixing resulted in proteins with no activity. However, an active Trm8/Trm82 heterodimer was synthesized when mRNAs of both Trm8 and Trm82 were co-translated [30]. These results strongly suggest that the association of the Trm8 and Trm82 subunits is translationally controlled in living cells.

It has been suggested that the manner of tRNA binding to eukaryotic tRNA m^7^G methyltransferase Trm8 is different from that in eubacterial TrmB [29]. This proposal is consistent with the results of biochemical studies. The major recognition sites of the yeast Trm8/Trm82 are the D- and T-stem structures. The G18-U55 and G19-C56 base pairs are not essential; however, disruption of the D- and T-loop interaction causes a severe decrease in methyltransfer activity [69]. In contrast, for eubacterial TrmB methylation, the most important site is located on the T-arm, and the tertiary base pairs in the tRNA three-dimensional core are not essential [23]. Thus, yeast Trm8/Trm82 has stricter recognition requirements for substrate tRNA than eubacterial TrmB. 

### 2.3. mRNA Cap m^7^G Methyltransferase

TrmB and mRNA cap-m^7^G methyltransferase (Abd1) have different targets for methylation. In the catalytic center of TrmB, the amino acid residues present differ totally from those in the catalytic center of Abd1. Thus, the reaction mechanism of TrmB is expected to differ from that of Abd1 [22,70]. The m^7^G methyltransferase of vaccinia virus mRNA capping enzyme is also a heterodimeric protein composed of the vD1 subunit and the vD12 subunit. The vD1 subunit constitutes an autonomous functional unit containing both RNA triphosphate and RNA guanyltransferase activities [71,72]. The m^7^G methyltransferase domain is heterodimerized with a stimulatory vD12 subunit [73,74]. An allosteric mechanism, whereby the vD12 subunit enhances the affinity of the catalytic vD1 subunit for AdoMet and the guanine acceptor, has been proposed. It has been shown that the catalytic subunits of vD1, as well as the yeast mRNA capping enzyme Abd1, are unrelated to Trm8. Furthermore, from sequence analysis, the noncatalytic subunit vD12 is not structurally related to Trm82. With respect to the relationship with rRNA m^7^G methyltransferase, neither Trm8 nor Trm82 has significant similarity to KgmB (the kanamycin-gentamicin resistance methylase, 16S rRNA (m^7^G1405) methyltransferase) from *Streptomyces tenebrarius*, a rRNA m^7^G methyltransferase associated with aminoglycoside resistance [36,37], other than the methyltransferase domain shared by numerous methyltransferases [75].

## 3. Physiological Functions

A large number of tRNA modifications have important roles in tRNA function [76]. In particular, tRNA modifications in the anticodon region play a major role in translation and growth [77]. The role of many tRNA modifications outside of the anticodon region are considered auxiliary to correct structure formation and fine tuning of the translation because it hardly appears as phenotypic defects [76,77]. Because of this, information about the role of the m^7^G46 modification in tRNA was limited for a long time, even though the modification is widely found in eubacteria and eukaryotes. However, clarification of the function of m^7^G46 in tRNA has begun over the past decade.

### 3.1. tRNA m^7^G46 Modification in Yeast

TrmB gene disruption in *E. coli* demonstrated no phenotypic defects [58]. Since both Trm8 and Trm82 are absolutely required to form m^7^G in yeast, a phenotype would be expected already in *trm8* or *trm82* single mutants and not requiring a double deletion [28]. Reference [57] actually shows the mentioned phenotype in *trm8* and *trm82* single mutants not only in double mutants. This conditional temperature sensitivity is the first example of a physiological function for m^7^G modification for tRNA. Since this report, various double gene deletion mutants of yeast have shown various phenotypes. Hypo-modified mature tRNA^Val^(AAC) deacylates and degrades rapidly in a double deletion mutant strain of *trm8* and *trm4* (*∆trm4∆trm8*), (Trm4 is a methyltransferase for 5-methyl cytidine at positions 34, 40, 48, and 49 in tRNA) at 37 °C, resulting in a temperature sensitive phenotype [78]. The temperature-sensitivity indicates that it relates to rapid tRNA decay (RTD) pathways. Deletion of *MET22*, which likely regulates 5′–3′ exonuclease Rat1 and Xrn1 activity indirectly, prevents tRNA^Val^(AAC) degradation in the *∆trm4∆trm8* strain. In this mutant strain, the tRNA^Val^(AAC) restores its aminoacylation and the growth defect is rescued. Thus, the RTD is mediated by Met22 and the 5′–3′ exonuclease Rat1 and Xrn1 [79]. Hypomodified tRNA, such as tRNA^Val^(AAC) from a *∆trm4∆trm8* mutant, is subject to degradation by RTD. However, stability of tRNA^Val^(AAC) is restored, upon overexpression *TEF1* and *VAS1*, which encode the elongation factor *eEF1A* and valyl-tRNA synthetase respectively, and which protect the hypomodified tRNA by direct interaction [80]. In addition, Maf1 indirectly affects maturation of the tRNA precursor [81]. In yeast, Maf1 is a negative regulator of Pol III, which mediates several signaling pathways [82,83]. Maf1 inhibits tRNA transcription via a mechanism dependent on phosphorylation and nuclear accumulation of Maf1, followed by physical association with Pol III in the tRNA genes. In a *∆trm4∆trm8* mutant, inhibition of Pol III activity reduces degradation of tRNA^Val^(AAC). Thus, reduction of tRNA transcription prevents degradation of hypomodified tRNA [80]. Surveillance of RNA quality and clearance of aberrant tRNA is important in all organisms.

### 3.2. tRNA m^7^G46 Modification in Thermophilic Eubacteria

In comparison with these eukaryotic enzymes, there is limited information about eubacterial enzymes. However, a part of the physiological importance of the m^7^G modification in tRNA has been revealed. We focused on characterization of a thermophilic *trmB* gene disruptant (*∆trmB*) strain of *Thermus thermophilus* HB8. *T. thermophilus* is an extreme thermophilic eubacteria and can live at wide range of temperatures, from 50 to 83 °C. A combination of distinct tRNA modifications, namely, Gm18, 5-methyl-2-thiouridine (m^5^s^2^U54), and m^1^A58, increase the tRNA melting temperature by nearly 10 °C compared with unmodified tRNA transcript [84,85,86]. These modifications do not affect translational fidelity under 65 °C, and the rate of the modification of tRNA is very low in cells cultured at 50 °C. The levels of these modified nucleosides in tRNA control translation via tRNA flexibility [87]. However, the mechanism by which modifications are controlled remained unknown until the beginning of the 21st century. When the *trmB* gene was disrupted, the introduction ratio of Gm18, m^5^s^2^U54, and m^1^A58 was dramatically changed, and the melting temperature of the hypo-modified tRNA decreased. In particular, degradation of tRNA^Phe^ and tRNA^Ile^ was detected. Furthermore, protein synthesis was depressed in the *∆trmB* strain at 70 °C, and it exhibits a severe growth defect at 80 °C [26]. Above 65 °C, m^7^G functions as a marker of precursor tRNA and increases the reaction rate of other modification enzymes. In contrast, at low temperature (50 °C), Ψ55 decreased the rate of formation of Gm, m^5^s^2^U54, and m^1^A58 and controls structural rigidity of tRNA [88]. In addition, the protein synthesis activity of the tRNA Ψ55 synthase (*truB*) disruptant strain was lower than that of the wild-type strain, and cold-shock proteins were absent in *∆truB* cells at low temperature [88]. Thus, the m^7^G46 and Ψ55 modifications work as an accelerator and a brake, respectively [6]. Therefore, these tRNA modification networks regulate the degree of modifications in response to temperature, and the response of the network to environmental change is very rapid. This is a typical strategy of eubacteria whose genome size is limited. 

### 3.3. Involvement of tRNA m^7^G46 Modification in Fungal Pathogenicity

The first report of the relationship between tRNA modification enzymes and fungal pathogenicity was by Takano et al., who showed that the tRNA m^7^G46 modification is required for plant infection by the phytopathogenic fungus *Colletotrichum lagenarium*, the cause of cucumber anthracnose [32]. *aph1* (Appressorial Penetration into Host) is required for efficient tRNA m^7^G46 modification in *C. lagenarium*, and experiments with *aph1* gene knockout mutants suggest that Aph1 is required for appressorium-mediated host invasion and also has important roles in resistance to several stresses including the basic defense response of the host plant. Given that in addition to m^7^G46 there are other tRNA modifications which are related to infection [6], tRNA modification and tRNA modification enzymes are likely be an important factor in the relationship between host and infectious organisms.

### 3.4. Involvement of tRNA m^7^G46 Methyltransferase in Diseases

Since tRNA modification regulates protein synthesis, there are several reports on the relationship between tRNA modification and genetic disease. METTL1 and WDR4 are the human homologues of Trm8 and Trm82, respectively. METTL1 has been initially identified as a substrate of protein kinase Bα and METTL1 phosphorylated at Ser27 is inactive [89]. Also, NSUN2 (NOP2/Sun domain family, member 2) is the mammalian ortholog of yeast Trm4 and has been identified as a substrate of protein kinase (Aurora-B) in HeLa cells [90]. As mentioned above, the yeast tRNA modification enzymes, Trm8 and Trm4, are required to protect tRNA from RTD. Moreover, in a yeast *∆trm8* strain, it has been observed that the cytotoxic effect of 5-fluorouracil (5-FU) is enhanced by heat stress [91]. 5-FU, which is a pyrimidine analogue, and is a widely used chemotherapeutic agent for treatment of solid cancerous tumours. However, increasing doses of 5-FU cause serious side effects. In order to reduce the side effects of 5-FU treatment, it is necessary to consider a new strategy. The observation of the influence of 5-FU in the yeast *∆trm8* strain leads to the hypothesis that these RTD-rerated modifying enzymes might affect the efficiency of 5-FU in human cancer cells. In fact, Okamoto et al. have shown that a knockdown strain of both NSUN2 and METTL1 genes drastically increases sensitivity to 5-FU in HeLa cells [92]. NSUN2 and METTL1 are phosphorylated by Aurora-B and protein Akt1/kinase Bα, respectively, and the enzymatic activities of NSUN2 and METTL1 are suppressed by phosphorylation. Therefore, constitutive overexpression of both dephosphorylated tRNA methyltransferases can repress 5-FU sensitivity. Thus, NSUN2 and METTL1 are involved 5-FU sensitivity in HeLa cells.

With respect to homologues of Trm82, in recent years, various important points have come out to light. In *Drosophila*, the gene *wh* (*wuho*), which has WD40 repeats, is a homologue of Trm82 and is essential for spermatogenesis and has a critical function in cellular differentiation for germline cells during gametogenesis [93]. In humans, the WDR4 gene mutation abolishes the m^7^G46 modification in tRNA and causes microcephalic primordial dwarfism characterized by facial dysmorphism, brain malformation, and severe encephalopathy with seizure. Amino acid substitution of WDR4 has been revealed as the cause mutation [94,95]

The molecular mechanism of microcephaly has not been well understood. In order to profile the m^7^G tRNA methylome in mouse embryonic stem cell (mESCs), two independent methods have been developed: m^7^G methylated immunoprecipitation sequencing (MeRIP-seq) and tRNA reduction and cleavage sequencing (TRAC-seq) [96]. In mESCs, the global m^7^G tRNA methylome is essential for appropriate translation of cell cycle genes and genes associated with brain malformation. Further to this observation, Mettl1 or Wdr4 knockout causes defective mESC self-renewal and neural lineage differentiation [96]. This study has clearly demonstrated the tRNA m^7^G methylome in mammals and shows the critical nature of METTL1 and WDR and m^7^G modification in regulation of stem cells and disease.

## 4. Perspective

Since m^7^G was found in tRNA, the genes encoding tRNA m^7^G methyltransferase have been identified in several organisms, and amino acid residues key to the reaction mechanism have been identified [22,25,29,57]. In addition, primitive quality control systems, resulting from tRNA m^7^G46 modification, have been demonstrated [26,78]. Furthermore, the physiological functions of m^7^G46 in tRNA have started to be determined over the past decade [92,93,94,95,96]. There seems to be little commonality between tRNA (m^7^G46) methyltransferase, rRNA m^7^G methyltransferase, and cap-m^7^G methyltransferase as far as the active centers are concerned. Recently, a reaction mechanism for TrmB has been proposed [25]. However, catalytic mechanism structural studies on AdoMet and/or the tRNA bound form of m^7^G methyltransferases are still necessary to fully understand the catalytic machinery. Also, genes encoding the tRNA m^7^G methyltransferase responsible for m^7^G at position 49 in archaeal tRNA and for anticodon m^7^G of mt tRNA or chloroplast tRNA have not yet been identified. From the viewpoint of m^7^G molecular evolution, it is important that reaction mechanisms of m^7^G methyltransferases from different substrates and various organisms are compared and analyzed.

## Figures and Tables

**Table 1:** *N*7-methylguanosine methyltransferase in tRNA.

Domains of Life	Organisms	Positions of m^7^G	Enzyme Names	Higher Order Structure	References
eubacteria	*E. coli*	46	TrmB	monomer	[21,22]
	*A. aeolicus*	46	TrmB	monomer	[23,24,25]
	*T. thermophilus*	46	TrmB	?	[26]
	*B. subtilis*	46	TrmB	homodimer	[27]
	*S. pneumoniae*	46	TrmB	homodimer	PDB: 1YZH
archaea	*T. acidophilum*	49 (tRNA^Leu^(UAG))	?	?	[7,20]
	*T. neutrophilus*	?	?	?	[7]
eukaryote	*S. cerevisiae*	46	Trm8/Trm82	heterodimer	[28,29,30]
	*S. cerevisiae*	5′ termini of pre-tRNA	Ceg1p	?	[31]
	*C. lagenarium*	46	Aph1	?	[32]
	human	46	METTL1/WDR4	?	[21,28]
	*C. reinhardtii*	37 (chloroplast tRNA^Leu^(UAG))	?	?	[16]
	*A. amurensis*	34 (mt tRNASer(GCU))	?	?	[17,19]
	*L. bleekeri*	34 (mt tRNASer(GCU))	?	?	[18,19]

Unidentified m^7^G positions, enzymes, and higher order structures are indicated by question marks.
